# Effects of a Moderate Increase in Dietary Starch Content and Partial Replacement of Dietary Protein Source with Canola Meal on Methane Emissions and Lactation Performance in Dairy Cows

**DOI:** 10.3390/ani16213482

**Published:** 2026-11-04

**Authors:** Mingkang Zhu, Tianyou Wu, Li Liu, Kui Li, Zhen Chen, Jie Wu, Miao Lin, Jiakun Wang, Ruihua Zhang, Xingkai Zhang, Huizeng Sun, Zihai Wei

**Affiliations:** 1 Ministry of Agriculture and Rural Affairs Key Laboratory of Dairy Cattles Genetic Improvement in Southern China, Bright Farming Co., Ltd., Shanghai 200436, China; mingkangzhu@zju.edu.cn (M.Z.); wutianyou@brightdairy.com (T.W.); wujie2@brightdairy.com (J.W.); zhangruihua@brightdairy.com (R.Z.); zhangxingkai@brightdairy.com (X.Z.); 2 MoE Key Laboratory of Molecular Animal Nutrition, Institute of Dairy Science, College of Animal Sciences, Zhejiang University, Hangzhou 310058, China; 22417075@zju.edu.cn (K.L.); jiakunwang@zju.edu.cn (J.W.); huizeng@zju.edu.cn (H.S.); 3 College of Animal Science and Technology, Yangzhou University, Yangzhou 225009, China; liliu9812@163.com (L.L.); cz18756682252@163.com (Z.C.); linmiao@yzu.edu.cn (M.L.)

**Keywords:** canola meal, dairy cows, methane emissions, protein source, starch content

## Abstract

This study aimed to evaluate the impact of moderately elevated dietary starch levels and the partial replacement of dietary protein source with canola meal (CM) on lactation performance, methane (CH_4_) emissions, ruminal fermentation parameters, and serum biochemical parameters in dairy cows. Initially, two in vitro gas production trials were conducted: Trial 1 involved diets formulated with starch levels of 24.0% (Low starch), 27.4% (Medium starch), and 30.8% (High starch) on a dry matter (DM) basis. In Trial 2, compared with a control diet without CM (CON_in_vitro_; 25.7% starch, the mean of 24.0% and 27.4%), a diet with moderately increased starch content and the inclusion of CM at 10.73% of DM (HSCM_in_vitro_; 29.1% starch, the mean of 27.4% and 30.8%) on a DM basis was formulated. In the subsequent in vivo experiment, thirty mid-lactation Holstein cows were randomly allocated to two groups: a control diet (CON, comprising 26.1% starch without CM, on a DM basis) or an experimental diet (HSCM, containing 28.9% starch with 7.68% CM, on a DM basis). In vitro results in Trial 1 showed that increasing dietary starch linearly decreased CO_2_ production (*p* < 0.01) and CH_4_ production (*p* = 0.03), but no significant differences between groups were detected. In Trial 2, a 3.4% increase in dietary starch combined with partial inclusion of CM showed a tendency to reduce CH_4_ (*p* = 0.06) and CO_2_ production (*p* = 0.08) compared to the CON diet. For the in vivo experiment, cows on the HSCM diet exhibited a tendency for greater dry matter intake (DMI; *p* = 0.06) and feed efficiency (milk yield/DMI; *p* = 0.09), and significantly improved milk yield (*p* < 0.01), energy-corrected milk (ECM; *p* < 0.01), and fat- and protein-corrected milk (FPCM; *p* < 0.01). The yields of milk fat (*p* < 0.01), protein (*p* < 0.01), lactose (*p* = 0.03), and total solids (*p* < 0.01) were also significantly improved, while the milk urea nitrogen (MUN) concentration was decreased (*p* < 0.01). The HSCM diet significantly decreased CH_4_ production by 14.1% (*p* = 0.02), as well as CH_4_ yield (*p* < 0.01) and CH_4_ emission intensity (*p* < 0.01) in terms of g/kg milk yield, ECM, and FPCM. Rumen fermentation parameters indicated lower molar proportions of isobutyrate and isovalerate in the HSCM group (*p* < 0.01), with no differences in other rumen parameters between groups. Additionally, the HSCM group exhibited a higher serum total protein (TP) concentration (*p* = 0.02) and a decreasing trend in blood urea nitrogen (BUN; *p* = 0.09). These findings demonstrate that a moderate increase in dietary starch content and partial replacement of dietary protein source with CM is an effective nutritional strategy for simultaneously improving lactation performance and mitigating CH_4_ emissions. However, as starch and CM were altered simultaneously in the in vivo experiment, the independent contribution of each factor cannot be determined and warrants further investigation.

## Simple Summary

Reducing methane emissions from dairy cows is important for sustainable milk production. This study investigated the effects of a moderate increase in dietary starch content and partial replacement of dietary protein source with canola meal on methane emissions and lactation performance in dairy cows. In vitro gas production assay showed that only a 3.4% (from 24.0% to 27.4% or 27.4% to 30.8% of dry matter) increase in dietary starch content was insufficient to effectively reduce rumen methane emissions. In contrast, a moderate 3.4% (from 25.7% to 29.1% of dry matter) increase in starch together with partial inclusion of canola meal as a dietary protein source more effectively reduced methane production. This strategy was further applied to lactating dairy cows, with a moderate 2.8% increase in dietary starch content and partial inclusion of canola meal (7.68% of the diet on a dry matter basis). The results demonstrated that this diet reduced methane production by 14.1% and markedly decreased methane emission intensity (g/kg energy-corrected milk), while dairy cows produced more milk and milk components. Our findings indicate that the combined strategy of moderately increasing dietary starch content and partial replacement of dietary protein source with canola meal is a practical nutritional strategy to reduce methane emissions and improve dairy farm productivity.

## 1. Introduction

Anthropogenic greenhouse gas (GHG) emissions from industrial and agricultural activities have disrupted the Earth’s climate balance and environmental stability, accelerating global warming [1]. Methane (CH_4_) is the second most important greenhouse gas, with a shorter atmospheric lifetime but a global warming potential (GWP) 82.5 times that of carbon dioxide (CO_2_) over a 20-year time horizon, and it contributes approximately 30% to global warming [2]. Therefore, mitigating CH_4_ emissions is of great significance for alleviating global warming in the short term. The livestock sector is one of the major sources of agricultural GHG emissions, contributing approximately one-third of global anthropogenic CH_4_ [3,4]. Owing to their specialized gastrointestinal tracts and microbial fermentation, ruminants produce substantial amounts of CH_4_ through the anaerobic fermentation of carbohydrates, accounting for 81% of total CH_4_ emissions from the livestock sector [5,6]. Furthermore, the energy lost as CH_4_ in ruminants represents approximately 2% to 12% of gross energy intake, leading directly to economic losses in feed costs [7]. Dairy cows play a vital role in the human food system by converting roughage unsuitable for human consumption into nutrient-rich milk. Although dairy farming is a major source of CH_4_ emissions, contributing approximately 30% of total GHG emissions from the global livestock sector [8], effective CH_4_ mitigation strategies should support or maintain the lactation performance of dairy cows to meet the growing global demand for dairy products.

Dietary formulation is an effective strategy for mitigating CH_4_ emissions without compromising lactational performance in dairy cows [9]. The carbohydrate composition of the diet directly influences the rumen fermentation patterns associated with CH_4_ production [6,10]. Fiber and starch, the two most important dietary carbohydrate fractions, play a key regulatory role in ruminal CH_4_ production [11]. Rumen bacteria, fungi, and protozoa are primarily responsible for fiber degradation, producing acetate and butyrate as well as substantial amounts of hydrogen (H_2_) during the breakdown of dietary fiber [12,13]. Most of the H_2_ released is utilized by methanogenic archaea for CH_4_ synthesis [14]. Focusing solely on inhibiting the H_2_ sink toward the methanogenic pathway may lead to an increased H_2_ partial pressure in the rumen, thereby inhibiting feed degradation [15,16]. Theoretically, ruminal starch degradation primarily promotes propionate fermentation, which acts as an effective H_2_ sink to reduce CH_4_ production [17]. However, a significant reduction in CH_4_ emissions is observed only when there is a wide starch level gradient between groups. Culbertson et al. [18] and Pirondini et al. [19] increased dietary starch content by 4% and 4.2%, respectively, and neither study observed a significant decrease in CH_4_ production or emission intensity (g/kg ECM) in dairy cows. Whereas Juckem et al. [20] reported that increasing dietary starch from 21% to 27% significantly reduced both CH_4_ production and emission intensity (g/kg ECM). Similarly, Aguerre et al. [21] found that increasing starch content by 3% and 6% did not significantly lower CH_4_ production, whereas a 9% increase in dietary starch content could significantly reduce CH_4_ emissions. These findings indicate that when dietary starch content is manipulated to mitigate CH_4_ emissions in dairy cows, a significant reduction in CH_4_ emissions is generally achieved only when the starch content is increased substantially, resulting in a relatively large starch content difference between diets.

Parallel to the starch-centric approach, considerable attention has been devoted to the dietary protein source as a modifiable determinant of CH_4_ emissions. Double-low rapeseed, characterized by low erucic acid content in the oil (<2%) and low glucosinolate levels in the meal (<30 μmol/g), is a common feed ingredient in dairy cow production [22]. The by-product obtained after solvent extraction of oil, referred to as double-low rapeseed meal or commonly canola meal (CM), serves as an economical alternative to soybean meal (SBM) in dairy cow diets [23,24]. In vivo studies have demonstrated that CM exhibits a lower CH_4_ production potential than SBM [25,26,27,28]. This may be attributed to the higher ruminal passage rate of CM compared with SBM, which reduces the amount of fermentable substrate available in the rumen [29,30]. Gidlund et al. [28] reported that, when CM was used as the protein source in the diet of Swedish Red cows, CH_4_ emission intensity (g/kg ECM) decreased significantly with increasing dietary crude protein (CP) concentration. Benchaar et al. [25] observed a linear reduction in CH_4_ emissions with increasing dietary inclusion of CM at the expense of SBM. Moreover, dietary inclusion of CM has been reported to improve DMI and lactational performance [30,31], thereby reducing CH_4_ emission intensity.

Increasing dietary starch is a common diet-formulation approach to maximize milk production of high-producing dairy cows, but feeding more starch in dairy diets may also increase the risk of ruminal acidosis, which is associated with a series of lactation performance and health problems in dairy cows [32,33]. A consensus has been reached in dairy cow diet formulation practice that dietary starch levels should not be excessively high, so as to avoid health and lactation problems. According to a 2023 nationwide survey conducted by the China Agricultural (Dairy) Research System, the dietary starch content for high-producing cows on modern large-scale farms mainly ranged from 24% to 31%. The average starch content for the top 50% was 29% and for the bottom 50% was 25.9%, and 75% of diets were formulated between 25% and 29%. This suggests that a relatively narrow and recognized range of dietary starch content has emerged within the dairy industry. Therefore, we intentionally selected an increase of approximately 3% in dietary starch content that stays within this commonly used range, rather than evaluating a broader range of starch contents that would deviate from typical commercial practice and potentially increase the risk of ruminal acidosis. However, such a moderate increase alone may not effectively reduce CH_4_ emissions. Therefore, the novelty of the present study lies in investigating whether combining this moderate starch increase with partial replacement of the dietary protein source with canola meal can simultaneously mitigate CH_4_ emissions and improve lactation performance, a strategy that has rarely been examined within the starch range typically used in the dairy industry. We hypothesized that this combined strategy would reduce CH_4_ emissions and improve milk production compared with a control diet without CM. The purpose of the current study is to investigate the effects of different starch content and CM inclusion on CH_4_ production in vitro, and the effects of moderately increasing dietary starch content and partially replacing dietary protein source with CM on lactation performance, CH_4_ emissions, ruminal fermentation parameters, and serum biochemical parameters in lactating dairy cows.

## 2. Materials and Methods

### 2.1. In Vitro Gas Production Assay

The in vitro gas production assay comprised two trials. In Trial 1, diets were formulated with starch levels of 24.0% (Low starch), 27.4% (Medium starch), and 30.8% (High starch) on a dry matter (DM) basis by adjusting the proportions of corn and were used as fermentation substrates. The ingredients and chemical composition of the diets in Trial 1 are presented in Table 1. In Trial 2, two diets were formulated on a DM basis and used as fermentation substrates: a control diet without CM (CON_in_vitro_; 25.7% starch, the mean of 24.0% and 27.4%), and an experimental diet with moderately increased starch content and the inclusion of CM at 10.73% of DM, with CM contributing 28.4% of the total dietary CP (HSCM_in_vitro_; 29.1% starch, the mean of 27.4% and 30.8%). The ingredients and chemical composition of the diets in Trial 2 are presented in Table 2. For each trial, 0.5 g of ground substrate based on DM was weighed and placed into a 120 mL serum bottle. Rumen fluid for Trial 1 was collected from three late-lactation dairy cows fitted with ruminal cannulas. These cows subsequently entered the dry period and were utilized again for rumen fluid collection in Trial 2. The artificial saliva solution was prepared according to the method of Menke et al. [34]. Before the morning feeding, rumen fluid was collected from multiple locations in the ventral sac of the rumen of donor cows through the ruminal cannula. The collected rumen fluid was filtered through four layers of cheesecloth, mixed with artificial saliva solution at a ratio of 1:2 (vol/vol), and continuously purged with CO_2_ to maintain anaerobic conditions. 75 mL of buffered rumen fluid was dispensed into each bottle, and the bottles were placed in a thermostatic incubator (IN150R; Huanmeibio, Suzhou, China) at 39 °C. Four replicates were prepared for each treatment, and four bottles containing no substrate served as blanks to correct for gas measurement. Thus, a total of 16 bottles (12 samples + 4 blanks) and 12 bottles (8 samples + 4 blanks) were incubated in Trial 1 and Trial 2, respectively. Headspace pressure of each bottle was measured, and gas samples were collected at 8, 24, and 48 h of incubation. The CH_4_ and CO_2_ concentrations were determined using a gas chromatograph (GC 2010; Shimadzu, Kyoto, Japan).

### 2.2. Animals, Feeds, and Experimental Design

The experiment was carried out at Xinghuo No.2 Dairy Farm in Shanghai from April to June, during which the average temperature ranged from 18 to 28 °C. Cows were housed in a tie-stall barn with a bedding area of approximately 3.5 m^2^ per cow and had free access to water. The bedding material consisted of wood shavings and was replaced daily. When the barn temperature exceeded 18 °C, fans were activated to provide an air velocity of 3 m/s across the cows. When the temperature surpassed 26 °C, overhead sprayers were also operated in a cycle of 2 min of spraying followed by a 1 min pause. Thirty lactating Holstein dairy cows (10 primiparous and 20 multiparous; body weight = 726.8 ± 58.2 kg, days in milk = 179.5 ± 10.1 d, milk yield = 40.1 ± 5.4 kg/d; mean ± SD) were used for this study. Cows were grouped into 15 blocks using a completely randomized block design based on parity, milk yield, and days in milk. One cow from each block was randomly assigned to one of two types of dietary treatments: (1) a control diet (CON, containing 26.1% starch on a laboratory-analyzed basis with no CM, on a DM basis) or (2) an experimental diet (HSCM, containing 28.9% starch on a laboratory-analyzed basis with 7.68% CM, on a DM basis). The diets were formulated to meet the nutrient requirements of lactating Holstein cows at mid-lactation, according to the NASEM (2021) recommendations [35]. The total mixed ration (TMR) was prepared three times daily using a vertical TMR mixer. Ingredients were added sequentially in the order of forages, concentrate supplements, and minerals. Additional water was incorporated to achieve a moisture content of 45–50% in the mixed ration, and the mixture was blended for approximately 15 to 20 min to ensure uniformity. The diets were provided to cows three times daily (0600, 1330, and 2100 h) to enable ad libitum intake with 5% to 10% refusal. The ingredients and chemical composition of the basal diet are shown in Table 3. The cows were milked three times daily (0600, 1300, and 2100 h). The experiment spanned 10 weeks, with the initial 2 weeks designated for dietary adaptation.

### 2.3. Feed Intake and Sample Analysis

The dry matter intake (DMI) of each cow was recorded for two consecutive days at two-week intervals. Approximately 500 g of two types of fresh TMR were collected weekly from six sampling points before the morning, noon, and evening feedings. The samples were dried in a forced-air oven at 65 °C for 48 h, and subsequently ground through a 1 mm sieve for further analysis. The DM (method 934.01), CP (method 988.05), EE (ether extract, method 954.02), starch (method 996.11), and Ash (method 942.05) contents were determined using the method of AOAC [36]. The neutral detergent fiber (NDF) and acid detergent fiber (ADF) contents were measured using an Ankom A200 fiber analyzer (Foss, Hillerød, Denmark) following the method of Van Soest et al. [37].

### 2.4. Milk Yield and Composition

Milk yield of each cow was recorded for two consecutive days each week, and 50 mL milk samples from each cow were collected at each milking using milk-sampling devices (Waikato Milking Systems NZ Ltd., Hamilton, New Zealand) on the second day. The milk samples were proportionally pooled based on yield and stored with added bronopol tablets (milk preservative; D & F Control System Inc., San Ramon, CA, USA) for subsequent analysis of milk protein, fat, lactose, total solids, urea nitrogen (MUN), and somatic cell counts (SCC) using a multifunction dairy analyzer (MilkoScan FT-120; FOSS, Hillerød, Denmark). Energy-corrected milk (ECM) was calculated using the following equation [38]: ECM (kg/d) = 0.327 × milk yield (kg/d) + 12.95 × fat yield (kg/d) + 7.2 × protein yield (kg/d), and fat- and protein-corrected milk (FPCM) was calculated using the following equation [39]: FPCM (kg/d) = milk yield (kg/d) × (0.337 + 0.116 × %fat + 0.06 × %protein).

### 2.5. In Vivo Gas Emissions Measurement

Gas emissions were measured using an Automatic Head-Chamber (AHC) according to the methods described previously at weeks 4 and 8 [40]. The AHC system consisted of a feed bin, a fan, an air filter, a collection pipe, an airflow meter, and a gas sampler. The feed bin was fully enclosed except for one side that allowed access for a single cow at a time, and it contained a trough where a small amount of bait feed was provided via a chute. The air inlet is located directly in front of the cows’ noses and mouths inside the bin, and is spatially separated from the fans and sprinklers above the barn to avoid interference. Gases from respiration and eructation were collected by the fan (300 L/s) and their volume measured by the airflow meter. The collected gas was drawn from the collection pipe at 2 L/min, passed through the air filter, and gas concentrations were recorded every second using a portable greenhouse gas analyzer (GGA-30p; Los Gatos Research, San Jose, CA, USA). The capture rate of CH_4_ emission was determined during the methane measurement period. A 300 mL volume of CH_4_ (purity > 99.9%) was instantaneously released into the feed trough to simulate eructation. The measured CH_4_ production was calculated from the concentration recorded by the AHC system, and the capture rate was derived as the ratio of the measured value to the actual injected volume. The capture rate of CH_4_ emission should be maintained between 90% and 110% to ensure proper performance of the AHC system under barn environmental conditions.

A three-day adaptation was conducted before the formal measurement. During this period, all cows were fed with bait feed and gradually adapted to the AHC until regular CH_4_ eructation peaks could be consistently detected. The regular occurrence of CH_4_ eructation peaks served as an additional physiological indicator that the cows remained in a relatively stable state, as stress or anxiety typically disrupts normal eructation patterns. The bait feed provided to each cow was kept below 3% DMI. During the formal measurement, two batches were measured repeatedly, and a total of eight visit time points (00:00, 03:00, 06:00, 09:00, 12:00, 15:00, 18:00, and 21:00) were measured for each cow in each batch to calculate the gas emissions over the entire day. Adjacent visits of each cow were at least 6 h intervals, with each visit lasting no less than 5 min. For every 5 dairy cows measured, a 2 min background gas measurement was conducted. During each 5 min measurement, methane eructation peaks were extracted for subsequent analysis, including peak duration, peak frequency, peak height, and integrated peak area. The average daily CH_4_ emission for each period was calculated as the mean of the two rounds within that period, and CH_4_ emission intensity was calculated based on contemporaneous measurements of milk yield, ECM, FPCM, and DMI. Throughout the measurement period, all cows exhibited normal feeding, rumination, and resting behaviors and showed no signs of stress or abnormality, such as head withdrawal or refusal to enter the chamber.

### 2.6. Blood Sample Collection and Analysis

Blood samples were obtained from the coccygeal vein at 3 h after morning feeding on day 0 (before treatment initiation) and on the final day of weeks 5 and 9, and placed into 10 mL vacuum tubes without anticoagulant. All blood samples were centrifuged at 3000× *g* for 15 min at 4 °C to isolate serum. The serum was then packaged and stored at −80 °C until analysis. The concentrations of glucose (GLU), non-esterified fatty acids (NEFA), β-hydroxybutyrate (BHBA), total protein (TP), albumin (ALB), blood urea nitrogen (BUN), aspartate aminotransferase (AST), alanine aminotransferase (ALT), superoxide dismutase (SOD) and total antioxidant capacity (TAC) were determined using an automated chemistry analyzer (BX-4000; Sysmex Corporation, Kobe, Japan) with commercial colorimetric kits (Medical System Biotechnology Co., Ltd., Ningbo, China).

### 2.7. Rumen Fluid Sample Collection and Analysis

Following completion of CH_4_ emission measurements for each cow and verification that the data contained no omissions or errors, rumen fluid was collected using an oral stomach tube on the final days of weeks 5 and 9 at 3 h after noon feeding. To avoid saliva contamination, the first 150 mL of rumen fluid was discarded, and approximately 50 mL of rumen fluid was then collected from each cow. The pH of rumen fluid was measured immediately using a pH meter (FE20-FiveEasy Plus™; Mettler Toledo Instruments Co. Ltd., Shanghai, China), and rumen fluid was packaged and stored at −80 °C for subsequent analysis. The ammonia nitrogen (NH_3_-N) was determined using a method described previously [41]. 1 mL of rumen fluid sample was thawed and thoroughly mixed with 20 μL of 85% orthophosphoric acid. The mixture was centrifuged at 13,000× *g* for 10 min at 4 °C, and the supernatant was filtered using a 0.22 μm syringe filter to obtain the sample for analysis. The volatile fatty acids (VFAs) were determined using a gas chromatograph (GC 2010, Shimadzu, Japan).

### 2.8. Statistical Analysis

All analyses were conducted using SAS (version 9.4; SAS Institute Inc., Cary, NC, USA). For in vitro data, including gas production and CH_4_ and CO_2_ emissions, intergroup comparisons were performed using PROC GLM. The statistical model was as follows:

Y_ij_ = μ +T_i_ + e_ij_ (1)

where Y_ij_ is the dependent variable, μ is the overall mean, T_i_ is the fixed effect of treatment, and e_ij_ is the residual error. Post hoc comparisons were performed using Tukey’s honestly significant difference (HSD) test, and linear and quadratic effects of treatment were tested using orthogonal polynomial contrasts for the starch level in vitro experiment data.

In the case of feeding trial data, which encompassed milk yield, milk component content and yield, ECM, FPCM, feed intake, gas emissions and their corresponding indicators, as well as rumen fermentation parameters and blood metabolites, a repeated-measures with PROC MIXED model was employed. The model incorporated treatment, week, and their interaction as fixed effects, while block was treated as a random effect, with cow serving as the subject for repeated measurements across weeks. Baseline values (d 0) were included as covariates for all variables, excluding rumen fermentation parameters, feed intake, gas emissions, and their corresponding indicators. The statistical model was as follows:

Y_ijk_ = μ +T_i_ +W_j_ + (TW)_ij_ + β·X_ijk_ + B_k_ + ε_ijk_ (2)

where Y_ijk_ is the dependent variable, μ is the overall mean, T_i_ is the treatment effect, W_j_ is the week effect, (TW)_ij_ is the treatment × week interaction, β·X_ijk_ is the covariate term (baseline value on day 0; included for all variables except rumen fermentation parameters, feed intake, enteric gas emissions, and their corresponding indicators), B_k_ is the random effect of block, and ε_ijk_ is the residual error. For the covariance structure for repeated measurements, we fitted five candidate structures: Variance Components (VC), Compound Symmetry (CS), First-order Autoregressive [AR(1)], Spatial Power [SP(POW)], and Unstructured (UN). Model selection was based on the Schwarz Bayesian information criterion.

Missing data were handled by the default restricted maximum likelihood (REML) procedure in PROC MIXED, without additional imputation. Abnormal data were identified at ± 2 SD from the group mean and verified against raw records. Values confirmed as technical errors were excluded; the remaining values between 2 and 2.5 SD were retained as biological extremes, while those >2.5 SD were excluded. Significance was established at *p* ≤ 0.05, and trends were noted at 0.05 < *p* < 0.10.

## 3. Results

### 3.1. In Vitro Gas Production

Table 4 displays the in vitro gas production in response to various levels of dietary starch. The rise in dietary starch content led to a linear decrease in total gas production from 196.5 mL/g DM to 160.5 mL/g DM (*p* < 0.01) and in CO_2_ production from 157.1 mL/g DM to 130.9 mL/g DM (*p* < 0.01). CH_4_ production also decreased linearly (*p* = 0.03), while the impact of starch on CH_4_ production tended to be significant (*p* = 0.07), and multiple comparison results showed that there was only a tendency of CH_4_ production reduction in the Low starch group compared with the High starch group (*p* = 0.06), with no difference between any other two groups. The proportions of CH_4_ (*p* = 0.73) and CO_2_ (*p* = 0.45) in the total gas remained unaffected by the dietary starch level.

Table 5 illustrates the in vitro gas production in response to moderately increased starch content and the partial substitution of soybean meal with canola meal. In comparison to the CON_in_vitro_ group, the HSCM_in_vitro_ diet showed a tendency to reduce CH_4_ production (*p* = 0.06) and CO_2_ production (*p* = 0.08), with no significant differences detected in total gas production (*p* = 0.12), CH_4_ proportion in total gas (*p* = 0.11), or CO_2_ proportion (*p* = 0.31).

### 3.2. DMI and Milk Production

**Figure 1:**
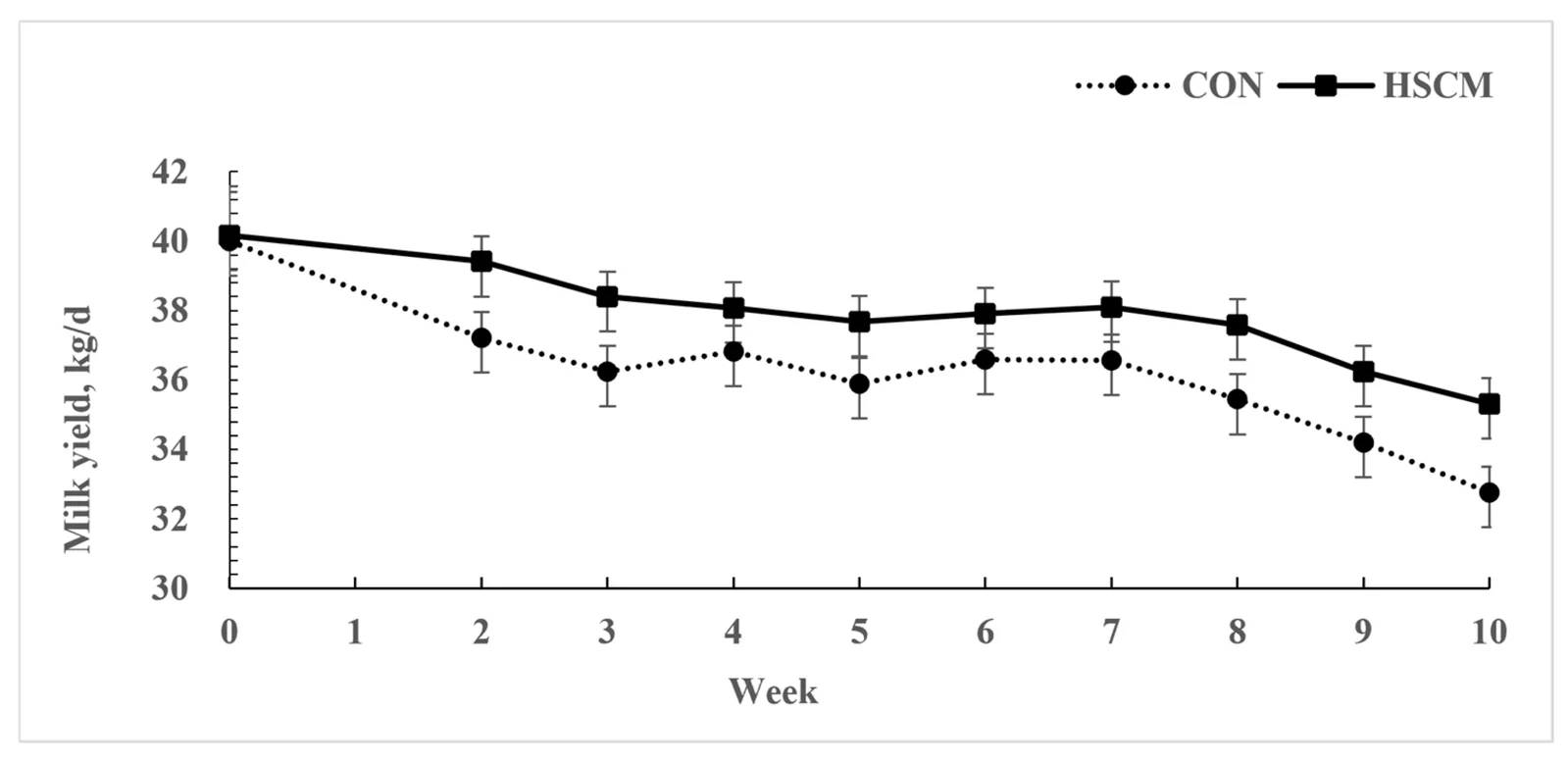
Weekly milk yield of dairy cows fed a control diet (CON, diet containing 26.1% starch with no CM) or an experimental diet (HSCM, diet containing 28.9% starch with 7.68% CM) during the experiment. *n* = 15 cows per group.

As shown in Table 6, compared with the CON group, the HSCM diet led to a tendency for higher DMI (24.9 vs. 24.1 kg/d; *p* = 0.06) and feed efficiency (milk yield/DMI; *p* = 0.09). Forage intake was lower in the HSCM group (9.04 vs. 9.59 kg/d; *p* < 0.01), whereas concentrate intake (15.8 vs. 14.5 kg/d; *p* < 0.01) and starch intake (7.18 vs. 6.28 kg/d; *p* < 0.01) were significantly greater. The HSCM diet significantly increased milk yield (37.4 vs. 35.6 kg/d; *p* < 0.01), ECM (41.4 vs. 39.3 kg/d; *p* < 0.01), and FPCM (38.1 vs. 36.3 kg/d; *p* < 0.01). From the second week onward, milk yield in the HSCM group exceeded that of the CON group, maintaining this effect superiority throughout the experiment (Figure 1). Furthermore, a significant time effect was observed for DMI, milk yield, ECM, and FPCM (*p* < 0.01). The MUN concentration in the HSCM group was lower than that in the CON group (13.7 vs. 14.3 mg/dL; *p* < 0.01), while the lactose percentage in the HSCM group showed a tendency to be lower than that in the CON group (5.09 vs. 5.13%; *p* = 0.06). The yields of fat (1.54 vs. 1.46%; *p* < 0.01), protein (1.29 vs. 1.22%; *p* < 0.01), lactose (1.91 vs. 1.83%; *p* = 0.03), and total solids (4.89 vs. 4.63%; *p* < 0.01) in the HSCM group were significantly higher than those in the CON group.

### 3.3. Gas Emissions

Gas emissions from dairy cows in response to increased starch content and partial replacement of soybean meal with canola meal are summarized in Table 7. Compared to the control diet, the HSCM diet significantly decreased CH_4_ production by 14.1% (474.6 vs. 552.7 g/d; *p* = 0.02). Furthermore, CH_4_ yield (19.2 vs. 23.5 g/kg DMI; *p* < 0.01) and CH_4_ emission intensity, expressed per kilogram of milk yield (12.7 vs. 15.3 g/kg; *p* < 0.01), ECM (11.1 vs. 13.7 g/kg; *p* < 0.01), and FPCM (12.1 vs. 14.9 g/kg; *p* < 0.01) were lower in the HSCM group. No significant difference in CO_2_ production was detected between the CON and HSCM groups (*p* = 0.31).

### 3.4. Rumen Fermentation Parameters

Rumen fermentation parameters are presented in Table 8. The HSCM diet did not alter ruminal pH (6.16 vs. 6.11; *p* = 0.29), NH_3_-N concentration (9.79 vs. 10.4 mg/dL; *p* = 0.53), total VFA concentration (130 vs. 133 mM; *p* = 0.40), the molar proportions of acetate (59.2 vs. 59.1%; *p* = 0.87), propionate (22.9 vs. 22.5%; *p* = 0.42), butyrate (14.8 vs. 14.9%; *p* = 0.77), valerate (1.50 vs. 1.55%; *p* = 0.27), or the acetate-to-propionate ratio (2.62 vs. 2.65; *p* = 0.63). The molar proportions of isobutyrate (0.64 vs. 0.72%; *p* < 0.01) and isovalerate (1.05 vs. 1.27%; *p* < 0.01) significantly decreased in the HSCM group.

### 3.5. Serum Biochemical Parameters

The serum biochemical parameters in dairy cows in response to increased starch content and partial replacement of dietary protein source with canola meal are shown in Table 9. The HSCM diet exhibited a tendency to decrease BUN concentration (4.98 vs. 5.31 mmol/L; *p* = 0.09) and significantly elevate TP concentration (70.5 vs. 68.2 g/L; *p* = 0.02). Additionally, lower TAC concentration (1.17 vs. 1.25 mmol/L; *p* < 0.01) was observed in the HSCM group. No significant differences (*p* > 0.05) were detected in other serum biochemical parameters between the CON and HSCM groups.

## 4. Discussion

### 4.1. In Vitro Methane Mitigation

The in vitro gas production technique simulates the fermentation of feeds in the rumen of ruminants, offering a rapid and convenient approach to investigate ruminal methanogenesis [42]. In an in vitro experiment, Yi et al. [43] observed a linear decrease in CH_4_ production (mL/g DM degradation) as the substitution ratio of corn stover by corn grain increased. Cone et al. [44] conducted in vitro fermentation using rumen fluid from cows fed diets with different starch contents, and found that the rumen fluid from donors fed a high-starch diet reduced CH_4_ production (mL/g organic matter) across all tested substrates. In Trial 1, CH_4_ production decreased linearly with increasing starch content, which is consistent with previous findings and our expectations. Further analysis showed that the increase in dietary starch level was accompanied by a decrease in NDF content from 31.3% to 25.3% in the High starch group, representing a reduction of approximately 6 percentage points. Fiber is an important substrate that contributes to gas production during ruminal degradation [45], and its reduction may have partially decreased the gas contribution from fiber fermentation. However, as a highly fermentable starch source with greater gas production potential than fiber [46], corn grain increased substantially in proportion from 26.1% to 39.3% in the High starch group. Therefore, the lower gas production observed in the High starch group cannot be simply attributed to a reduction in total fermentable substrate. A possible explanation is that rapid fermentation of the High starch diet led to a marked decline in ruminal pH, which may have inhibited fibrolytic bacteria and consequently reduced the efficiency of NDF degradation, ultimately decreasing total gas production. This interpretation is consistent with previous reports that high-starch diets reduce pH and suppress fiber degradation [47]. Nevertheless, since pH dynamics and NDF digestibility were not directly measured during fermentation in the present study, this explanation remains speculative, and the relative contributions of each factor warrant further investigation.

However, the magnitude of starch increment required to achieve a statistically significant reduction remains a critical question. Previous studies have reported that a significant reduction in CH_4_ emissions is achieved when dietary starch content is increased by 6% to 9% [20,21]. In Trial 1, we evaluated two incremental levels: a moderate increase of 3.4 percentage units (from 24.0% to 27.4% of DM) and a larger increase of 6.8 percentage units (from 24.0% to 30.8% of DM). Interestingly, the 3.4% starch increase showed no notable effect on CH_4_ production, whereas the 6.8% increase tended to reduce CH_4_ production, suggesting a dose-dependent trend under the in vitro conditions. This finding suggests that while increasing dietary starch has the potential to mitigate CH_4_, achieving a significant reduction may require an increment exceeding 6.8% under the present in vitro conditions—or that starch alone is insufficient and must be combined with other methane-mitigating strategies.

Given that dietary starch levels cannot be excessively increased in practice due to the constraints of rumen health and production costs [48], two dietary starch levels (25.7% and 29.1% on a DM basis) were selected for the subsequent in vivo experiment, aligning with the requirements of high-producing dairy cows on modern large-scale farms in China (24–31%) and representing typical commercial conditions. Because an increase of about 3% in dietary starch levels may be insufficient to substantially reduce CH_4_ emissions, CM was further introduced to partially replace the dietary protein source. CM has been reported to reduce methanogenesis through multiple mechanisms, including a higher ruminal passage rate that limits fermentable substrate availability [29,30] and a slower protein degradation rate that improves energy-nitrogen synchrony [49]. Paula et al. [50] reported that the replacement of SBM with CM in the diet tended to reduce CH_4_ concentration and CH_4_ production (mL/g DM). In Trial 2, CH_4_ production in the HSCM group was reduced by 17%, which was 3.65 to 9.55 percentage points greater than the reduction achieved by increasing 3.4% starch content alone (from 24% to 27.4% of DM, or 27.4% to 30.8% of DM), consistent with our expectations that CM and starch exert a synergistic effect on methane mitigation.

### 4.2. In Vivo Lactation Performance

Based on the in vitro findings, we further evaluated the in vivo effects of the HSCM diet on lactation performance. DMI in dairy cows is influenced by dietary starch levels and protein sources [51,52]. Malekkhahi et al. [53] reported a 1.2 kg/d increase in DMI when dietary starch content was increased from 22% to 32%. The physical fill resulting from the higher NDF content of the control diet (Table 3), particularly the higher forage NDF associated with its greater forage proportion, may also have contributed to its lower DMI [52]. In addition, DMI increased linearly with increasing CM inclusion in a previous study [25]. Similarly, Sánchez-Duarte et al. [54] also found that completely replacing SBM with CM and increasing dietary starch content to 27% enhanced DMI in dairy cows. The tendency for increased DMI in the HSCM group may be attributed to moderate starch levels that raise energy density and CM’s slower proteolysis [29], which may together contribute to improving ruminal energy–nitrogen synchrony and DMI. However, this proposed mechanism remains speculative and has not been directly measured in the present study.

As a rapidly fermentable carbohydrate, starch serves as a major energy source for lactating dairy cows, and increasing dietary starch content has been shown to markedly increase milk yield and FPCM by supplying additional energy for milk synthesis [18,55]. However, the milk yield response to increased dietary starch is not always consistent, as Piccioli-Cappelli reported no significant improvement in milk yield when starch content was elevated during early and late lactation. More digestible energy offered by increased starch, combined with the enhanced DMI, is crucial for sustaining lactation and likely contributed in part to the higher milk yield, ECM, and FPCM observed in the HSCM group in the current study. Additionally, the contribution of CM warrants consideration, as it potentially improves nitrogen utilization via an optimized methionine supply [49], a higher proportion of rumen-undegradable protein (RUP; Table 3), and improved energy-nitrogen synchrony [49]. These findings from the mechanistic perspective are corroborated by previous dose–response studies in which Benchaar et al. [25] found a linear increase in milk yield and ECM with higher levels of CM replacement of SBM. However, the effect of CM inclusion on milk yield has not been consistent across studies, which may be attributed to differences in basal diet type [54], CM inclusion level, and dietary CP content [28].

Dairy cows fed high-starch diets are often more prone to reduced milk fat concentration, which may be attributed to dilution effects from increased milk production, prioritization of nutrient allocation to adipose tissue, or alterations in the rumen environment and mammary fatty acid synthesis [56,57]. In the present study, milk fat concentration was maintained while milk fat yield increased significantly as a result of higher milk production, suggesting that the HSCM diet did not negatively affect fat production. The physically effective NDF (peNDF) provided by the forage components could support rumination and salivary buffer production, which help to maintain ruminal pH and milk fat concentration. However, because particle size distribution was not measured in this study, the contribution of peNDF to the stable ruminal pH remains to be investigated in future studies. Additionally, the higher methionine content in CM may have contributed to maintaining ruminal pH [58], thereby counteracting the acidogenic risk associated with the 2.8 percentage-unit increase in dietary starch. Because low ruminal pH is a known trigger for milk fat depression through altered biohydrogenation pathways and the production of trans-10, cis-12 conjugated linoleic acid [59], the relatively stable pH observed in the HSCM group further supports the absence of negative effects on fat synthesis.

It is well established that increasing dietary starch content can enhance milk protein and lactose yields [57,60]. In addition, previous studies have shown that CM could serve as an effective protein source in dairy cow diets to improve milk protein yield [61,62]. Consistent with the above findings, the yields of milk protein and lactose in the HSCM group were significantly increased in the present study, which may also be attributable to the higher milk output. Furthermore, the significant decrease in MUN concentration indicates that more nitrogen was partitioned into milk protein synthesis rather than excreted as urea, reflecting an overall improvement in dietary nitrogen utilization efficiency. Collectively, the higher milk yield, ECM, FPCM, and milk component yields observed in the HSCM group are consistent with expectations. This highlights the importance of optimizing both starch levels and protein sources in diet formulation for lactating dairy cows.

### 4.3. Rumen Fermentation and Methane Mitigation

Multiple studies have shown that increasing dietary starch content can reduce CH_4_ production by promoting the propionate fermentation pathway [63,64]. However, the magnitude of this effect appears to depend on the extent of starch increase, as not all studies have observed significant reductions. For instance, Culbertson et al. [18] and Pirondini et al. [19] increased dietary starch content by 4% and 4.2%, respectively, but neither detected a significant decrease in CH_4_ production or emission intensity (g/kg ECM) in dairy cows. These inconsistent findings suggest that a moderate increase in starch alone may be insufficient to achieve meaningful methane mitigation, highlighting the need for complementary strategies within the limited adjustable range of dietary starch.

Contrary to expectations, the molar proportion of ruminal propionate in the HSCM group did not increase in parallel with the reduction in CH_4_ emissions, nor was there a detectable decrease in the acetate-to-propionate ratio. This is consistent with Mc Geough et al. [65], who reported that the reduction in CH_4_ emissions may not be directly associated with changes in fermentation end-products as measured by static VFA molar proportions. However, this observation warrants careful interpretation. One important consideration is that ruminal volatile fatty acids (VFA) molar proportions represent a dynamic equilibrium between production, absorption, and liquid outflow, rather than serving as a direct reflection of their production rates [21]. When starch fermentation is accelerated, the generation of acetate, propionate, and butyrate may increase simultaneously, leaving the proportional contribution of propionate relatively unchanged. More importantly, propionate is the most rapidly absorbed VFA across the rumen epithelium, with an absorption rate substantially greater than that of acetate and butyrate [66]. Thus, even if net propionate production increased in response to starch fermentation, rapid absorption could prevent its accumulation in the rumen pool, thereby maintaining a stable molar proportion. Nevertheless, without direct measurements of ruminal liquid dilution rate or net VFA production, these interpretations remain speculative and require further investigation in future studies.

The lower molar proportions of isobutyrate and isovalerate in the HSCM group provide additional clues regarding the mechanisms underlying CH_4_ reduction. These branched-chain volatile fatty acids (BCVFAs) are end-products of branched-chain amino acids (BCAAs) fermentation in the rumen [67]. Compared with CM, SBM contains higher contents of isoleucine, leucine, and valine [25,68], and these BCAAs in SBM are also more extensively degraded in the rumen [29], which might explain the lower molar proportions of isobutyrate and isovalerate observed in the HSCM group due to a high proportion of SBM in CON. As BCVFAs may influence NDF degradation and CH_4_ production [69], this change might be associated with the lower CH_4_ emissions observed in the HSCM group. In addition, the high ruminal passage rate of CM might limit substrate availability in the liquid and particulate phases for methanogens, thereby reducing CH_4_ production [25,70,71]. However, these proposed mechanisms remain speculative and require further investigation.

The enhanced methane reduction observed with the combined diet may be partially attributed to improved synchrony of energy and nitrogen supply in the rumen, although direct evidence was not obtained in this study. Starch fermentation provides carbon skeletons and ATP for the propionate pathway, which is traditionally viewed as a key hydrogen sink that competes with methanogenesis. However, the competitive utilization efficiency of the propionate pathway might be constrained by the availability of rumen-degradable protein (RDP) for microbial protein synthesis. Compared with soybean meal, CM has a slower RDP degradation rate [29,49], which might better match the temporal pattern of starch fermentation, thereby enhancing the competitive utilization of hydrogen via the propionate pathway. This energy–nitrogen synchrony and CM’s methane-mitigating mechanisms may contribute to explaining why a moderate starch increase of only 2.8 percentage units, when combined with CM, achieved a 14.1% reduction in methane production, an effect comparable to or greater than that reported with substantially larger starch increments alone [20,21]. However, these mechanistic interpretations remain speculative and require confirmation through future studies that directly measure ruminal fermentation dynamics, microbial communities, and hydrogen metabolism. Notably, the reduction in CH_4_ emission intensity in the HSCM group exceeded that of CH_4_ production by 2.86 to 4.85 percentage points, a difference reflecting the additional dilution effect from improved milk production.

### 4.4. Systemic Metabolic Indicators

TP serves as a key indicator of protein nutritional status in dairy cows and is positively correlated with milk yield [72,73]. High-starch diets increase the supply of rumen-fermentable carbohydrates and improve the amino acid utilization efficiency in dairy cows [74], whereas CM promotes more efficient nitrogen metabolism in the rumen and provides a higher proportion of RUP [29,49]. Together, these mechanisms may explain the higher TP concentration observed in the HSCM group. This enhanced N utilization is further supported by the lower BUN concentration in the HSCM group, as BUN is a product of the ruminal nitrogen cycle and is associated with nitrogen utilization efficiency [75,76].

High milk production is often accompanied by increased metabolic load, which may affect oxidative balance. Ma et al. [77] reported that high-concentrate diets could induce subacute ruminal acidosis and decrease TAC in mammary venous blood. In the present study, the significant decrease in blood TAC observed in the HSCM group may reflect a combined effect of increased metabolic load from higher milk production, elevated dietary starch content [77], and potential oxidative challenges associated with unsaturated fatty acids in CM. As all measured values remained within the normal physiological ranges reported for dairy cows, this change is more likely to reflect a metabolic adaptation to higher energy intake rather than clinically relevant oxidative stress. However, the possibility that this decline reflects a mild increase in systemic oxidative challenge should not be excluded, and further investigation with additional oxidative stress markers and extended observation periods is warranted to better elucidate this change and its potential implications.

## 5. Conclusions

In vitro results suggest that a notable ruminal methane reduction required a large starch increase (6.8%), whereas a moderate increase (3.4%) was insufficient unless combined with canola meal. These results were validated in vivo: moderate starch (2.8%) with canola meal (7.68% DM) improved DMI, milk yield and milk component yields, reduced methane by 14.1%, and lowered emission intensity (g/kg ECM) more markedly. The mechanisms underlying these benefits and the independent contributions of starch versus canola meal remain unknown and require further investigation. In conclusion, a moderate starch increase combined with canola meal as the dietary protein source is a practical strategy to simultaneously improve lactation performance and reduce enteric methane emissions in dairy cows within the narrow starch range typically employed in commercial operations.

## Figures and Tables

**Table 1:** Ingredients and chemical composition of experimental diets for in vitro gas production in Trial 1 (DM basis, %).

Item	Experimental Treatments ^1^		
	Low Starch	Medium Starch	High Starch
Ingredient			
Alfalfa hay	10.52	10.53	10.54
Oaten hay	5.32	5.32	5.33
Corn silage	22.38	22.39	22.41
Corn grain	26.13	32.64	39.33
Wheat bran	8.16	4.17	0.00
Soybean hulls	7.20	3.60	0.00
Soybean meal	15.98	17.04	18.09
Sodium bicarbonate	1.18	1.18	1.19
Minerals and vitamins premix ^2^	0.55	0.55	0.55
Salts	0.55	0.55	0.55
Dicalcium phosphate	0.25	0.25	0.25
Calcium carbonate	1.23	1.23	1.23
Potassium Bicarbonate	0.18	0.18	0.18
Magnesium oxide	0.36	0.36	0.36
Chemical composition ^3^			
OM	95.4	95.6	95.9
CP	16.2	16.3	16.4
Starch	24.0	27.4	30.8
EE	3.34	3.40	3.47
NFC	44.6	47.6	50.7
NDF	31.3	28.3	25.3
ADF	17.4	15.5	13.5
Ash	4.60	4.37	4.13
NE_L_, MJ/kg DM	7.32	7.41	7.57

^1^ Low starch, diet with 24.0% starch; Medium starch, diet with 27.4% starch; High starch, diet with 30.8% starch; ^2^ Formulated to provide for one kilogram of premix: 1,360,000 IU vitamin A; 360,000 IU vitamin D; 9000 mg vitamin E; 3 g Cu; 18 g Zn; 10.5 g Mn; 88 mg Se; 185 mg I; 95 mg Co; ^3^ NE_L_ estimated using AMTS 4.20.0 (based on CNCPS v6.5); CP, Starch, EE, NDF, ADF and Ash were measured; OM and NFC calculated by difference (OM = 100 − Ash; NFC = 100 − CP − NDF − EE − Ash).

**Table 2:** Ingredients and chemical composition of experimental diets for in vitro gas production in Trial 2 (DM basis, %).

Item	Experimental Treatments ^1^	
	CON_in_vitro_	HSCM_in_vitro_
Ingredient		
Alfalfa hay	10.81	10.71
Oaten hay	5.46	5.41
Corn silage	22.98	22.77
Corn grain	28.76	35.80
Wheat bran	3.56	0.00
Soybean hulls	4.07	0.00
Soybean meal	20.34	10.61
Canola meal	0.00	10.73
Sodium bicarbonate	1.10	1.10
Minerals and vitamins premix ^2^	0.51	0.51
Salts	0.51	0.51
Dicalcium phosphate	0.23	0.23
Calcium carbonate	1.15	1.14
Potassium Bicarbonate	0.16	0.16
Magnesium oxide	0.34	0.34
Chemical composition ^3^		
OM	95.4	95.6
CP	17.1	17.1
Starch	25.7	29.1
EE	3.31	3.41
NFC	46.2	48.3
NDF	28.8	26.8
ADF	16.1	14.7
Ash	4.59	4.38
NE_L_, MJ/kg DM	7.36	7.41

^1^ CON_in_vitro_, diet with 25.7% starch and without CM; HSCM_in_vitro_, diet with 29.1% starch and the inclusion of CM at 10.73% of DM; ^2^ Formulated to provide for one kilogram of premix: 1,360,000 IU vitamin A; 360,000 IU vitamin D; 9000 mg vitamin E; 3 g Cu; 18 g Zn; 10.5 g Mn; 88 mg Se; 185 mg I; 95 mg Co; ^3^ NE_L_ estimated using AMTS 4.20.0 (based on CNCPS v6.5); CP, Starch, EE, NDF, ADF and Ash were measured; OM and NFC calculated by difference (OM = 100 − Ash; NFC = 100 − CP − NDF − EE − Ash).

**Table 3:** Ingredients and nutrient composition of experimental diets in dairy cows (DM basis, %).

Item	Experimental Treatments ^1^	
	CON	HSCM
Ingredient		
Alfalfa hay	8.70	8.84
Oaten hay	5.92	5.10
Corn silage	25.23	22.41
Brewer’s grain	4.20	3.12
Beet pulp	4.91	3.78
Corn grain, ground	21.7	22.04
Corn grain, steam flaked	4.79	9.69
Solvent soybean meal	16.2	11.6
Soybean hulls	1.2	0
Extruded soybean meal	0.5	0.47
Canola meal	0	7.68
Cottonseed meal	1.4	0
Calcium salts of fatty acids	0.77	0.76
Palm fat powder	0.81	0.80
Sodium bicarbonate	1.01	1.02
Minerals and vitamins premix ^2^	0.47	0.48
Salts	0.47	0.47
Dicalcium phosphate	0.21	0.21
Calcium carbonate	1.05	1.06
Potassium Bicarbonate	0.15	0.16
Magnesium oxide	0.31	0.31
DM	48.3	48.5
Chemical composition ^3^		
OM	92.3	92.4
CP	17.1	16.9
RUP (% CP)	34.0	36.3
Starch	26.1	28.9
EE	4.30	4.46
NFC	40.6	41.3
NDF	30.4	29.8
ADF	18.6	18.9
Ash	7.65	7.56
NE_L_, MJ/kg DM	7.41	7.49

^1^ CON, diet containing 26.1% starch with no CM; HSCM, diet containing 28.9% starch with 7.68% CM; ^2^ Formulated to provide for one kilogram of premix: 1,360,000 IU vitamin A; 360,000 IU vitamin D; 9000 mg vitamin E; 3 g Cu; 18 g Zn; 10.5 g Mn; 88 mg Se; 185 mg I; 95 mg Co; ^3^ RUP and NE_L_ estimated using AMTS 4.20.0 (based on CNCPS v6.5); CP, Starch, EE, NDF, ADF and Ash were measured; OM and NFC calculated by difference (OM = 100 − Ash; NFC = 100 − CP − NDF − EE − Ash).

**Table 4:** In vitro gas production in response to different dietary starch contents ^1^.

Items	Experimental Treatments ^2^			SEM	*p*-Value		
	Low Starch	Medium Starch	High Starch		Starch	Linear	Quadratic
Total gas, mL/g DM	196.5 ^a^	189.4 ^a^	160.5 ^b^	5.20	<0.01	<0.01	0.12
CH_4_, mL/g DM	31.15	28.83	24.98	1.65	0.07	0.03	0.71
CH_4_, % total gas	15.85	15.21	15.50	0.58	0.73	0.68	0.53
CO_2_, mL/g DM	157.1 ^a^	151.4 ^a^	130.9 ^b^	3.45	<0.01	<0.01	0.12
CO_2_, % total gas	79.98	80.06	81.66	1.01	0.45	0.27	0.55

^1^ *n* = 4 replicates per group; ^2^ Low starch, diet with 24.0% starch; Medium starch, diet with 27.4% starch; High starch, diet with 30.8% starch; ^a,b^ Means within the same row with different superscripted letters differ significantly (*p* < 0.05).

**Table 5:** In vitro gas production in response to moderately increased starch content and partial replacement of dietary protein source with canola meal (CM) ^1^.

Items	Experimental Treatments ^2^		SEM	*p*-Value
	CON_in_vitro_	HSCM_in_vitro_		
Total gas, mL/g DM	143.5	136.6	2.66	0.12
CH_4_, mL/g DM	15.59	12.94	0.83	0.06
CH_4_, % total gas	10.86	9.47	0.52	0.11
CO_2_, mL/g DM	120.4	110.9	3.22	0.08
CO_2_, % total gas	83.91	81.14	1.75	0.31

^1^ *n* = 4 replicates per group; ^2^ CON_in_vitro_, diet with 25.7% starch and without CM; HSCM_in_vitro_, diet with 29.1% starch and the inclusion of CM at 10.73% of DM.

**Table 6:** Lactation performance in dairy cows in response to increased starch content and partial replacement of dietary protein source with canola meal (CM) ^1^.

Item	Experimental Treatments ^2^		SEM	*p*-Value		
	CON	HSCM		Treat	Time	Treat × Time
DMI, kg/d	24.1	24.9	0.43	0.06	<0.01	0.19
Forage intake, kg/d	9.59	9.04	0.16	<0.01	<0.01	0.21
Concentrate intake, kg/d	14.5	15.8	0.26	<0.01	<0.01	0.18
Starch intake, kg/d	6.28	7.18	0.12	<0.01	<0.01	0.17
Milk yield, kg/d	35.6	37.4	0.55	<0.01	<0.01	0.86
Feed efficiency ^3^	1.63	1.69	0.04	0.09	<0.01	0.04
Milk composition						
Fat, %	4.11	4.17	0.05	0.35	<0.01	0.06
Protein, %	3.42	3.45	0.02	0.19	<0.01	0.58
Lactose, %	5.13	5.09	0.02	0.06	<0.01	0.51
Total solids, %	13.01	13.15	0.06	0.12	<0.01	< 0.01
MUN ^4^, mg/dL	14.3	13.7	0.18	<0.01	<0.01	0.02
SCC ^5^, 10^3^/mL	224.2	167.0	45.3	0.38	0.52	0.76
Yield, kg/d						
Fat	1.46	1.54	0.02	<0.01	<0.01	0.94
Protein	1.22	1.29	0.02	<0.01	<0.01	0.66
Lactose	1.83	1.91	0.03	0.03	<0.01	0.73
Total solids	4.63	4.89	0.06	<0.01	<0.01	0.11
ECM ^6^	39.3	41.4	0.56	<0.01	<0.01	0.11
FPCM ^7^	36.3	38.1	0.52	<0.01	<0.01	0.13

^1^ *n* = 15 cows per group; ^2^ CON, diet containing 26.1% starch with no CM; HSCM, diet containing 28.9% starch with 7.68% CM; ^3^ Feed efficiency = ECM yield (kg/d)/DMI (kg/d); ^4^ MUN, milk urea nitrogen; ^5^ SCC, somatic cell count; ^6^ ECM (kg/d) = 0.327 × milk yield (kg/d) + 12.95 × fat yield (kg/d) + 7.2 × protein yield (kg/d); ^7^ FPCM = milk yield (kg/d) × (0.337 + 0.116 × %fat + 0.06 × %protein).

**Table 7:** Gas emissions in dairy cows in response to increased starch content and partial replacement of dietary protein source with canola meal (CM) ^1^.

Item	Experimental Treatments ^2^		SEM	*p*-Value		
	CON	HSCM		Treat	Time	Treat × Time
CH_4_ emissions						
g/d	552.7	474.6	28.5	0.02	0.98	0.09
g/kg milk	15.3	12.7	0.67	<0.01	0.63	0.07
g/kg ECM	13.7	11.1	0.54	<0.01	0.07	0.10
g/kg FPCM	14.9	12.1	0.59	<0.01	0.08	0.10
g/kg DMI	23.5	19.2	1.09	<0.01	0.09	0.21
CO_2_ emissions, kg/d	12.0	11.1	0.62	0.31	0.02	0.29

^1^ *n* = 15 cows per group; ^2^ CON, diet containing 26.1% starch with no CM; HSCM, diet containing 28.9% starch with 7.68% CM.

**Table 8:** Ruminal fermentation parameters in dairy cows in response to increased starch content and partial replacement of dietary protein source with canola meal (CM) ^1^.

Item	Experimental Treatments ^2^		SEM	*p*-Value		
	CON	HSCM		Treat	Time	Treat × Time
pH	6.11	6.16	0.05	0.29	<0.01	0.35
NH_3_-N, mg/dL	10.4	9.79	0.72	0.53	0.28	0.91
Total VFA, mM	133	130	3.32	0.40	0.06	0.21
Molar proportion, mmoL/100 mmoL						
Acetate	59.1	59.2	0.44	0.87	<0.01	0.25
Propionate	22.5	22.9	0.39	0.42	<0.01	0.99
Butyrate	14.9	14.8	0.36	0.77	0.41	0.50
Isobutyrate	0.72	0.64	0.02	<0.01	0.07	0.06
Valerate	1.55	1.50	0.03	0.27	<0.01	< 0.01
Isovalerate	1.27	1.05	0.06	<0.01	0.27	0.18
Acetate/propionate	2.65	2.62	0.06	0.63	<0.01	0.93

^1^ *n* = 15 cows per group; ^2^ CON, diet containing 26.1% starch with no CM; HSCM, diet containing 28.9% starch with 7.68% CM.

**Table 9:** Serum biochemical parameters in dairy cows in response to increased starch content and partial replacement of dietary protein source with canola meal (CM) ^1^.

Item ^3^	Experimental Treatments ^2^		SEM	*p*-Value		
	CON	HSCM		Treat	Time	Treat × Time
GLU, mmol/L	2.48	2.55	0.05	0.35	<0.01	<0.01
NEFA, mmol/L	0.195	0.198	0.006	0.80	<0.01	0.24
BHBA, mmol/L	0.77	0.76	0.03	0.81	0.14	<0.01
TP, g/L	68.2	70.5	0.76	0.02	0.02	0.65
ALB, g/L	26.4	26.0	0.35	0.43	<0.01	0.58
BUN, mmol/L	5.31	4.98	0.14	0.09	<0.01	0.80
AST, U/mL	109.8	106.2	4.51	0.58	<0.01	0.02
ALT, U/mL	29.6	29.2	0.65	0.69	0.02	0.63
SOD, U/mL	105.9	102.6	2.20	0.32	<0.01	0.06
TAC, mmol/L	1.25	1.17	0.01	< 0.01	<0.01	0.58

^1^ *n* = 15 cows per group; ^2^ CON, diet containing 26.1% starch with no CM; HSCM, diet containing 28.9% starch with 7.68% CM; ^3^ GLU = glucose; NEFA = non-esterified fatty acids; BHBA = β-hydroxybutyrate; TP = total protein; ALB = albumin; BUN = blood urea nitrogen; AST = aspartate aminotransferase; ALT = alanine aminotransferase; SOD = superoxide dismutase; TAC = total antioxidant capacity.

## Data Availability

The data presented in this study are available on request from the corresponding author.

## Abbreviations

The following abbreviations are used in this manuscript:

ADF	Acid detergent fiber
ALB	Albumin
ALT	Alanine aminotransferase
AST	Aspartate aminotransferase
BUN	Blood urea nitrogen
CH_4_	Methane
CM	Canola meal
DM	Dry matter
DMI	Dry matter intake
ECM	Energy-corrected milk
EE	Ether extract
FPCM	Fat- and protein-corrected milk
GLU	Glucose
MUN	Milk urea nitrogen
NDF	Neutral detergent fiber
NE_L_	Net energy for lactation
NFC	Non-fibrous carbohydrate
NH_3_-N	Ammonia nitrogen
OM	Organic matter
RUP	Rumen-undegradable protein
SBM	Soybean meal
SCC	Somatic cell counts
SOD	Superoxide dismutase
TAC	Total antioxidant capacity
TP	Total protein
VFAs	Volatile fatty acids
